# The Prognostic Value of Inflammatory Indices in Predicting Poor Postoperative Outcomes in Isolated Coronary Artery Bypass Graft Surgery

**DOI:** 10.7759/cureus.43120

**Published:** 2023-08-08

**Authors:** Sameh Alagha, Ökkeş H Miniksar, Muhammed N Polat, Mehmet Kara, Yeşim Şenaylı

**Affiliations:** 1 Department of Cardiovascular Surgery, Yozgat Bozok University, Yozgat, TUR; 2 Department of Anesthesiology and Reanimation, Yozgat Bozok University, Yozgat, TUR

**Keywords:** chronic obstructive pulmonary disease, creatinine, biomarker, inflammation, coronary artery bypass

## Abstract

Background:This study aimed to analyze the predictive effect of various inflammatory indices and inflammatory biomarkers on prognosis after coronary artery bypass grafting (CABG).

Methods:In this retrospective observational study, data were recorded from 99 patients who underwent isolated elective CABG between January 2019 and June 2021 and met the inclusion criteria. The patients were divided into two main groups according to the postoperative clinical results: “favorable outcome” and “poor outcome.” Preoperative inflammatory parameters, inflammatory indices (such as systemic inflammation index (SII), neutrophil/lymphocyte ratio (NLR), derived NLR (dNLR)), and clinical variables were compared between the groups.

Results:Poor postoperative outcomes developed in 31 (31.3%) patients. In the univariate analysis, white blood cell count (p=0.008), neutrophil count (p=0.002), SII (p=0.018), NLR (p=0.003), and dNLR (p=0.003) were found to be significant predictors for poor outcomes. In the multivariate analysis, only the presence of chronic obstructive pulmonary disease (COPD) (OR=8.765; 95% CI 1.308-58.702; p=0.025) and high creatinine levels (OR=1.049; 95% CI 1.005-1.094; p=0.027) were independent risk factors for poor outcomes. Optimal cut-off values were 603.08 (areas under the curve (AUC)=0.632, p=0.036) for SII, 2.34 (AUC=0.669, p=0.007) for NLR, and 1.76 (AUC=0.667, p=0.008) for dNLR.

Conclusion:SII, NLR, dNLR, and inflammatory markers, such as white blood cell and neutrophil counts, are feasible markers for predicting poor outcomes following CABG procedures. These parameters may aid in the development of early therapeutic interventions to improve patient outcomes.

## Introduction

Coronary artery disease is one of the leading causes of death worldwide. One of the definitive treatments for coronary artery disease is coronary artery bypass grafting (CABG). Despite significant advances in cardiac surgery, emerging unfavorable postoperative outcomes affect morbidity and mortality [1,2]. Therefore, it is critical to identify individuals at high risk of adverse outcomes prior to surgery [3,4].

Atherosclerosis, which is considered an inflammatory process, plays a critical role in the pathogenesis of cardiovascular disease. The physiopathology of atherosclerosis is influenced by circulating neutrophils, platelets, and lymphocytes, which have essential roles in chronic inflammatory processes. However, increasing evidence in the literature suggests that an increased inflammatory response is responsible for various complications after cardiac surgery [5-7]. This evidence has increased interest among researchers in the subject of inflammation and clinical outcomes following cardiac surgery.

Previous studies have shown that various inflammatory indices, such as systemic inflammation index (SII), neutrophil/lymphocyte ratio (NLR), and inflammatory biomarkers (i.e., C-reactive protein (CRP) level), can predict mortality and morbidity after cardiac surgery [3-8]. However, in addition to the established risk factors, there is still a need in daily practice for an ideal, readily measured marker, such as a hematological parameter, to predict clinical outcomes. As a result, we hypothesized that all these indices could be independent predictors of substantial adverse clinical outcomes following CABG.

This study aimed to assess and analyze the predictive impact of various inflammatory indices and inflammatory biomarkers on prognosis following CABG.

## Materials and methods

Study design

**Figure 1:**
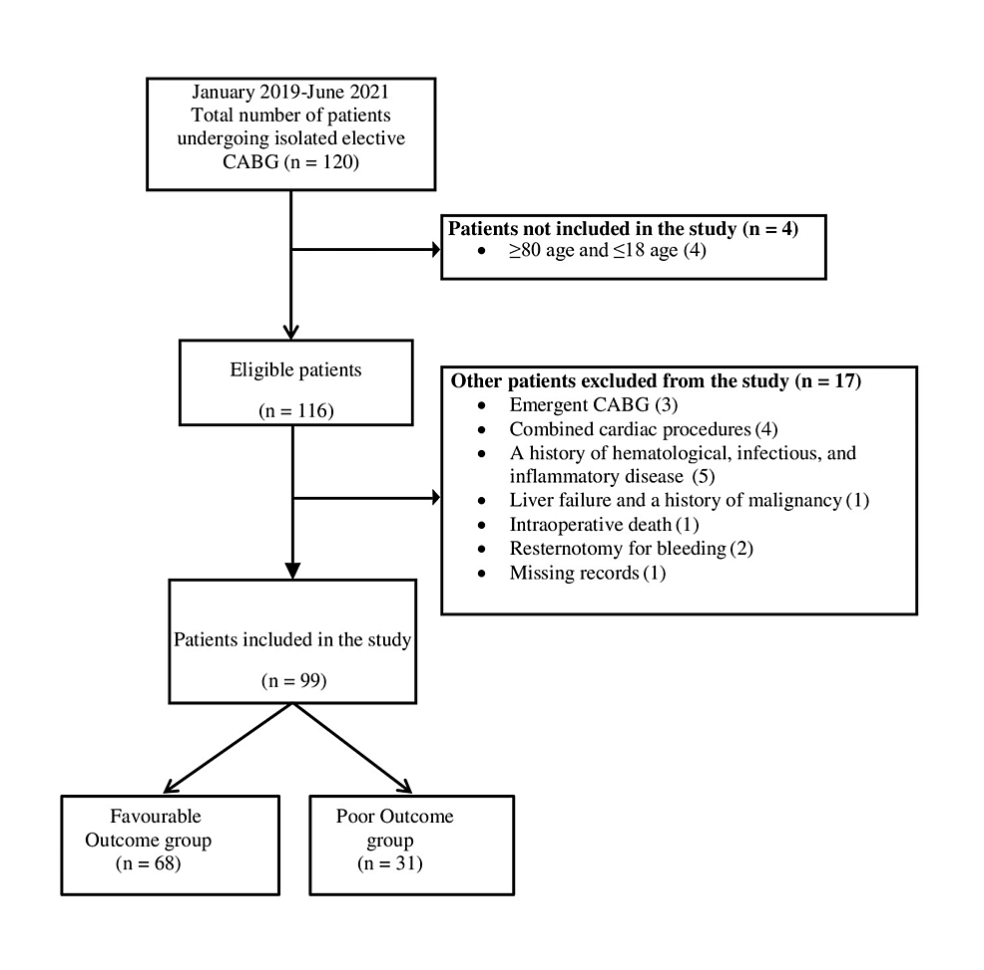
Flow diagram of the study

This retrospective, observational, single-center study was approved by the Clinical Research Ethics Committee of Yozgat Bozok University in accordance with the Declaration of Helsinki (No: 189_2021.08.25_04). This study was conducted by reviewing the data of 120 patients who underwent isolated elective CABG at our cardiovascular surgery department between January 2019 and June 2021. Inclusion criteria were patients between 18 and 80 years of age and isolated elective on-pump or off-pump CABG. Exclusion criteria were as follows: (1) emergent CABG; (2) patients 18 years of age or younger; (3) combined cardiac procedures; (4) a history of hematological, infectious, or inflammatory disease; (5) liver failure or a history of malignancy; (6) intraoperative death; (7) resternotomy for bleeding; and (8) missing records. Following the application of the exclusion criteria, the study included 99 participants (Figure 1).

Study participants

In addition to demographic data, the preoperative hematological parameters, procedure type, comorbidities, European System for Cardiac Operative Risk Evaluation (EuroSCORE) II, left ventricular ejection fraction (LVEF), pulmonary arterial pressure (PAP), coronary bypass time, intensive care unit (ICU) stay, length of hospital stay, and postoperative results were recorded. Data were retrospectively collected from electronic medical hospital records. The same experienced team of heart surgeons performed all surgeries.

The patients were divided into two groups according to the postoperative clinical results: “favorable outcome” and “poor outcome.” The patients’ postoperative data were analyzed for the development of poor outcomes, which were defined by the presence of one or more of the following: major adverse cardiovascular event (MACE), need for mechanical ventilation (MV) over 24 hours, new-onset renal failure, sepsis, and death. MACE was defined as the presence of one of the following: myocardial ischemia with ST-segment elevation, cerebrovascular incident or transient ischemic attack, cardiac arrest, low cardiac output syndrome. The latter was diagnosed when the placement of an intraoperative intra-aortic balloon pump (IABP) was required due to hemodynamic instability and/or the use of inotropic support (involving at least two of the following: dopamine, dobutamine, or epinephrine) was needed to facilitate weaning from cardiopulmonary bypass or within the first 24 hours in the ICU [4,5].

Laboratory measurements

Laboratory parameters obtained from venous blood samples taken from each patient within the day preceding surgery were recorded from the hospital medical records. The following inflammatory indices were calculated from whole blood assays: SII ([neutrophils×platelets]/lymphocytes), NLR (neutrophils/lymphocytes), derived NLR (dNLR) (neutrophils/[lymphocytes-neutrophils]), NLPR (neutrophil/[lymphocytes×platelets]), CRP/Alb ratio (CRP/albumin), CRP/L (CRP/lymphocytes), PLR (platelets/lymphocytes), and MPR (mean platelet volume/platelets).

Statistical analysis

Sample Size Estimation

The main examined association was the predictive effect of various inflammatory biomarkers on prognosis after CABG surgery. For the sample size calculation, we considered the estimated profiles of areas under the curve (AUC) of the association between predictors and poor outcomes following CABG. We estimated that a minimum sample size of 80 patients was needed to estimate an AUC of 75%, considering an error of 10%, a confidence interval of 95%, and a power of 80%. Sample size calculation was performed using the Power Analysis and Sample Size (PASS) 11.0 software program (Stata Corp. LP, College Station, TX, USA).

Outcome Analyses

Statistical analysis was performed using SPSS for Windows version 25.0 (IBM Corp., Armonk, NY, USA). Categorical data are presented as n and frequency, while continuous data are presented as mean ± standard deviation and median (interquartile range; 25th-75th percentile). The Kolmogorov-Smirnov and Shapiro-Wilk tests were used to assess the normality distribution. In cross tables, Fisher’s exact test was performed when more than 20% of the expected values were smaller than 5 or at least one of the values was smaller than 2. Univariate analysis was performed, and all significant variables (chronic obstructive pulmonary disease (COPD), urea, creatinine, white blood cell (WBC) and neutrophil counts, SII, NLR, and dNLR) were included in the multivariate logistic analysis. A backward stepwise multivariate logistic regression analysis was conducted to analyze the predictors of poor postoperative outcomes. The Hosmer-Lemeshow test for goodness-of-fit was used to determine the calibration validation and discrimination of this regression analysis. Receiver operating characteristic (ROC) curve analysis was performed to identify the predictive value of parameters and inflammatory indices for poor postoperative outcomes, and the AUC was calculated. A p-value of <0.05 was considered statistically significant.

## Results

Demographic and clinical characteristics

A total of 99 patients, including 72 women (72.7%) and 27 men (27.3%), who underwent CABG and met the study criteria were included (Table 1). The mean age of the patients was 62.36 ± 10.09 years. Poor outcomes developed in 31 (31.3%) patients and mortality was observed in 10 (10.1%). Most (n=69) patients underwent on-pump bypass surgery, while 30 underwent off-pump surgery. The type of surgery did not differ significantly between the groups (p = 0.110).

The most common comorbidities were hypertension (60.6%), diabetes mellitus (49.5%), and hyperlipidemia (22.2%), with no significant differences between the groups. COPD was significantly higher in the poor outcome group (n=6) compared with the favorable outcome group (n = 2) (p = 0.005). The mean total bypass time was 249.28 ± 100.84 for all patients. The median ICU length of stay was 6 (4-8) days. As reported in Table 1, the poor outcome group had significantly longer ICU stays (p = 0.005) and hospital stays (P = 0.017) than the favorable outcome group. A total of 10 (10.1%) patients required IABP.

**Table 1:** Comparison of demographic and clinical features between groups Values are reported as the mean (SD) and median (interquartile range) for continuous and percentage for categorical variables. All statistically significant values are reported in bold. 1) Yates’s correction for continuity; 2) Fisher’s exact test; 3) Independent t-test; 4) Mann-Whitney U test. COPD: chronic obstructive pulmonary disease; LVEF: left ventricular ejection fraction; PAP: pulmonary arterial pressure; EuroSCORE II: European System for Cardiac Operative Risk Evaluation

			Overall (n =99)	Favorable Outcome (n = 68)	Poor Outcome (n = 31)	p
Age (years)			62.36 (10.09)	61.10 (10.77)	65.13 (8.07)	0.065^3^
Gender	Female		72 (72.7)	48 (70.6)	24 (77.4)	0.479^1^
	Male		27 (27.3)	20 (29.4)	7 (22.6)	
Surgery type	On-pump		69 (69.7)	44 (64.7)	25 (80.6)	0.110^1^
	Off-pump		30 (30.3)	24 (35.3)	6 (19.4)	
Hypertension			60 (60.6)	40 (58.8)	20 (64.5)	0.591^1^
Diabetes mellitus			49 (49.5)	35 (51.5)	14 (45.2)	0.560^1^
Hyperlipidemia			22 (22.2)	14 (20.6)	8 (25.8)	0.562^1^
COPD			8 (8.1)	2 (2.9)	6 (19.4)	0.005^2^
EuroSCORE II			1.11 [0.78 to 1.43]	1.08 [0.76 to 1.385]	1.19 [0.79 to 1.72]	0.123^4^
LVEF (%)			55 [45 to 58]	55 [45 to 60]	50 [40 to 55]	0.830^4^
PAP			25 [20 to 30]	25 [20 to 30]	26 [23 to 30]	0.332^4^
Operation time (min)			249.28 (100.84)	241.16 (98.25)	267.10 (105.74)	0.237^3^
ICU stay duration (days)			6 [4 to 8]	5 [3 to 7]	8 [5 to 16]	0.005^4^
Hospital stay duration (days)			10 [8 to 15]	10 [7.5 to 13]	14 [8 to 20]	0.017^4^
Mortality			10 (10.1)	0 (0.0)	10 (32.3)	<0.001^2^
Use of IABP		No	89 (89.9)	68 (100.0)	21 (67.7)	<0.001^2^
		Yes	10 (10.1)	0 (0.0)	10 (32.3)	

Biochemical and total blood count parameters and inflammatory indices

When laboratory parameters were examined, the poor outcome group had significantly higher urea (p = 0.007), creatinine (p = 0.001), WBC (p = 0.021), and neutrophil (P = 0.005) counts. The following inflammatory indices were significantly higher in the poor outcome group: SII (p = 0.036), NLR (p = 0.007), dNLR (p = 0.008), NLPR (p = 0.003), and MPR (p = 0.042). In contrast, there were no significant differences in other laboratory parameters or inflammatory indices between the groups (Table 2).

**Table 2:** Comparison of laboratory variables between groups Values are reported as the mean (SD) and median (interquartile range) for continuous. All statistically significant values are reported in bold. 1) Mann-Whitney U test, 2) Independent t-test CRP: C-reactive protein; WBC: white blood cell; MPV: mean platelet volume; SII: systemic immune-inflammation index; NLR: neutrophil to lymphocyte ratio; dNLR: derived neutrophil to lymphocyte ratio; NLPR: neutrophil to lymphocyte x platelet ratio; PLR: platelets/lymphocytes ratio; MPR: mean platelet volume/platelet count ratio

Variables	Overall (n =99)	Favorable Outcome (n = 68)	Poor Outcome (n = 31)	p
Biochemical parameters				
Urea (mg/dL)	32.96 [25.89 to 38.95]	31.13 [25.14 to 36.91]	35.65 [30.82 to 41]	0.007^1^
Creatinine (mg/dL)	0.84 [0.74 to 0.99]	0.81 [0.7 to 0.92]	0.98 [0.8 to 1.3]	0.001^1^
Albumin (g/dL)	4.22 [3.99 to 4.44]	4.23 [4.01 to 4.44]	4.2 [3.77 to 4.36]	0.267^1^
CRP (mg/dL)	4.72 [2.18 to 14.7]	4.45 [1.87 to 10.65]	8.38 [2.52 to 17.9]	0.141^1^
Total blood count				
WBC (×10^9^ L)	7.98 [6.87 to 9.93]	7.69 [6.755 to 9.19]	8.93 [7.69 to 11.92]	0.021^1^
Neutrophils (×10^9^ L)	5.18 [4.2 to 7.24]	4.925 [4.175 to 6.21]	5.94 [4.86 to 8.97]	0.005^1^
Lymphocytes (×10^9^L)	2.09 (0.77)	2.14 (0.78)	1.98 (0.75)	0.355^2^
Platelets (×109 L)	241.74 (63.01)	249.00 (61.74)	225.81 (63.83)	0.089^2^
MPV (fL)	10 [9.5 to 10.56]	10 [9.4 to 10.58]	10 [9.6 to 10.5]	0.347^1^
Inflammatory indices				
SII	606.83 [478.14 to 890.61]	566.16 [478.39 to 748.00]	828.25 [461.65 to 1084.09]	0.036^1^
NLR	2.52 [1.97to 3.75]	2.31 [1.93 to 3.06]	3.58 [2.34 to 5.15]	0.007^1^
dNLR	1.92 [1.49 to 2.59]	1.74 [1.45 to 2.14]	2.34 [1.68 to 3.30]	0.008^1^
NLPR	0.01 [0.00 to 0.01]	0.00 [0.00 to 0.01]	0.01 [0.00to 0.02]	0.003^1^
CRP/alb	1.11 [0.50 to 3.35]	1.01 [0.43 to 2.53]	2.39 [0.61 to 4.68]	0.119^1^
CRP/L	2.49 [1.16 to 7.38]	2.30 [0.90 to 4.86]	2.81 [1.45 to 11.55]	0.159^1^
PLR	126.90 (44.32)	127.62 (42.99)	125.30 (47.82)	0.810^2^
MPR	0.04 [0.03 to 0.05]	0.04 [0.03 to 0.05]	0.04 [0.03 to 0.05]	0.042^1^

Risk factors for poor postoperative outcome using univariate and multivariate analysis

The results of univariate and multivariate logistic regression analysis for poor postoperative outcomes in patients who underwent CABG are presented in Table 3. In univariate analysis, the following preoperative variables were found to be significant predictors of poor outcome: COPD (OR = 7.920; 95% CI 1.498-41.870; p = 0.015), urea (OR = 0.1050; 95% CI 1.012-1.089; p = 0.009), creatinine (OR=22.334; 95% CI 3.037-164.236; P = 0.002), WBC count (OR = 1.253; 95% CI 1.060-1.481; P = 0.008), neutrophil count (OR=1.389; 95% CI 1.125-1.714; P = 0.002), SII (OR = 1.001; 95% CI 1.000-1.003; P = 0.018), NLR (OR = 1.541; 95% CI 1.162-2.043; P = 0.003), and dNLR (OR=2.074; 95% CI 1.274-3.377; P = 0.003). Multivariate analysis revealed that COPD (OR=8.765; 95% CI 1.308-58.702; P = 0.025) and high creatinine levels (OR = 1.049; 95% CI 1.005-1.094; P = 0.027) were independent predictors of poor postoperative outcomes. Other important variables (creatinine, WBC and neutrophil counts, SII, NLR, and dNLR) were not independent predictors of poor postoperative outcomes. The Hosmer-Lemeshow goodness-of-fit test indicated a well-calibrated model (P = 0.281).

**Table 3:** Univariate and multivariate regression analysis for predicting poor postoperative outcomes Hosmer and Lemeshow Test 9.77, p = 0.281, Nagelkerke R Square = 0.459, p < 0.001. Independent variables: COPD, urea, creatinine, WBC, neutrophils, SII, NLR, dNLR. All statistically significant values are reported in bold. CI: confidence interval; COPD: chronic obstructive pulmonary disease; WBC: white blood cell; SII: systemic immune-infammation index; NLR: neutrophil to lymphocyte ratio; dNLR: derived neutrophil to lymphocyte ratio.

Variables	Univariate analysis					Multivariate analysis					
	OR	(95% CI)			p	OR	(95% CI)				p
		Lower	Upper				Lower			Upper	
Age	1.045	0.997		1.096	0.069	--		--	--		--
Female	1.429	0.531		3.846	0.480	--		--	--		--
On-pump surgery	2.273	0.819		6.305	0.115	--		--	--		--
Diabetes mellitus	1.288	0.549		3.020	0.561	--		--	--		--
COPD	7.920	1.498		41.870	0.015	8.765		1.308	58.702		0.025
Urea	1.050	1.012		1.089	0.009	--		--	--		--
Creatinine	22.334	3.037		164.239	0.002	1.049		1.005	1.094		0.027
WBC	1.253	1.060		1.481	0.008	--		--	--		--
Neutrophils	1.389	1.125		1.714	0.002	--		--	--		--
SII	1.001	1.000		1.003	0.018	--		--	--		--
NLR	1.541	1.162		2.043	0.003	--		--	--		--
dNLR	2.074	1.274		3.377	0.003	--		--	--		--

Predictive accuracy of laboratory parameters for poor postoperative outcomes

**Figure 2:**
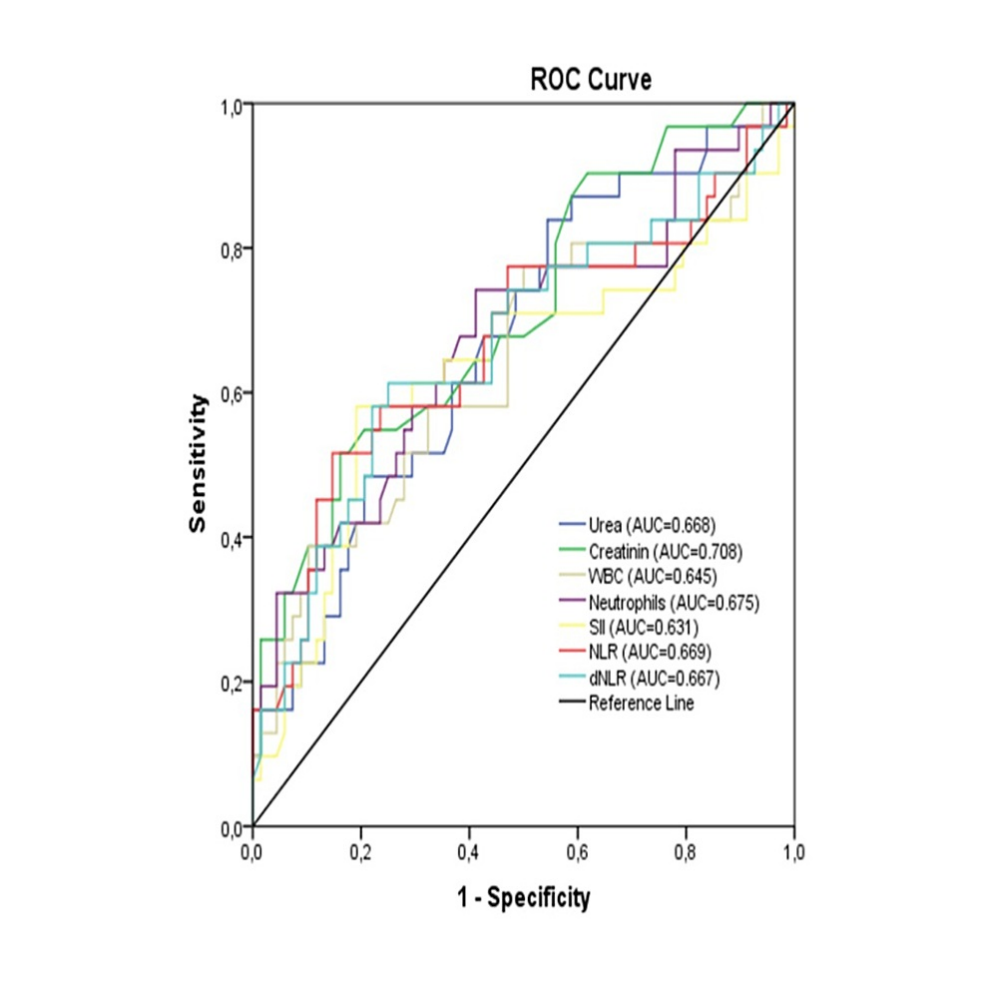
Receiver operating characteristic curve analysis

In the ROC analysis for poor postoperative outcomes in patients after CABG, the optimal cut-off values were 30.91 mg/dL for urea (AUC = 0.669, P = 0.007), 0.708 for creatinine (AUC = 0.669, P = 0.001), 7.73 for WBC count (AUC = 0.646, P = 0.021), 5.21 for neutrophil count (AUC = 0.676, P = 0.005), 603 for SII (AUC = 0.632, P = 0.036), 2.34 for NLR (AUC = 0.669, P = 0.007) and 1.76 for dNLR (AUC = 0.667, P = 0.008) (Table 4, Figure 2). However, ROC analysis for MPR did not reach a significant level in predicting poor postoperative outcomes after CABG.

**Table 4:** ROC curve analysis in predicting the poor postoperative outcome of cardiac bypass patients All statistically significant values are reported in bold AUC: area under the curve; CI: confidence interval; WBC: white blood cell; SII: systemic immune-infammation index; NLR: neutrophil to lymphocyte ratio; dNLR: derived neutrophil to lymphocyte ratio.

Variables	AUC	Cut-off point	Sensitivity (%)	Specificity (%)	95% CI		p
					Lower	Upper	
Urea	0.669	30.91	74.2	51.5	0.555	0.783	0.007
Creatinine	0.708	0.81	67.7	54.6	0.597	0.820	0.001
WBC	0.646	7.73	74.2	51.5	0.522	0.770	0.021
Neutrophils	0.676	5.21	67.7	61.3	0.556	0.796	0.005
SII	0.632	603.08	71.0	55.9	0.500	0.763	0.036
NLR	0.669	2.34	77.4	52.9	0.544	0.794	0.007
dNLR	0.667	1.76	74.2	51.5	0.544	0.790	0.008

## Discussion

Unfavorable outcomes have been reported following CABG; therefore, it is essential that early, rapid, simple, and reliable testing be available for patients who are at increased risk of developing adverse outcomes. Risk scoring systems and biomarkers are used to predict poor events in the preoperative period. Many scoring systems have been reported in the literature to predict mortality after cardiac surgery, such as EuroSCORE II, and the Society of Thoracic Surgeons score [9]. Ünal et al. [3] reported that EuroSCORE was not predictive for major poor events. In the current study, despite overall low EuroSCORE II results with nonsignificant differences between the groups, we had relatively high mortality rates. Since each institution and patient population may have unique risk factors and characteristics that can influence outcomes, it is crucial to emphasize that EuroSCORE is a general risk assessment tool for cardiac surgery, and it may not capture specific factors contributing to poor outcomes in certain patient groups. Factors such as systemic inflammation, immune response, and inflammatory biomarkers, may play a significant role in predicting postoperative outcomes, particularly in patients with chronic inflammatory conditions such as diabetes. Hence, many novel biomarkers for prognosis in cardiac surgery have recently been reported.

Earlier studies identified systemic inflammation and immune activation as risk factors for poor outcomes following cardiac surgery. SII and NLR are the most studied biomarkers in the diagnosis and prognosis of cardiovascular diseases, and they can be obtained from routine blood tests. In the current study, we found that the dNLR value (OR = 2.074, P = 0.003) was more predictive than other inflammatory indices. Another study reported that increased postcardiotomy NLR was the most predictive factor of major adverse events following CABG [8]. In their study, Ozer et al. stated that postoperative NLR could be useful in predicting ICU stay after CABG and managing treatment [10]. According to Ünal et al., preoperative NLR is an independent factor for predicting early mortality following CABG (OR = 6.47, 95% CI 1.18-35.38, P = 0.031) [3]. In the same study, the threshold NLR value for mortality was determined to be 2.81 (AUC=0.72, sensitivity=75%, specificity=67%). Furthermore, the authors stated that a low lymphocyte count, rather than an increased neutrophil count, was associated with mortality. The neutrophil count may be utilized as a marker of enhanced inflammatory response and may play a key role in controlling systemic inflammation [3,10-13]. However, in our study, elevated neutrophil count, rather than lymphocyte count, was a significant predictor of poor outcomes following CABG. Moreover, following ROC analysis, increased neutrophil count was found to be a significant predictor of poor outcomes (AUC = 0.67, sensitivity = 67%, specificity = 61%).

SII is another crucial inflammatory parameter that was examined in our study. The SII value is obtained by multiplying the NLR value times the platelet count. Prior studies have indicated SII as an independent predictor of poor outcomes in cardiac patients [4,5,11,12]. In our study, following univariate regression analysis, SII was found to be a significant predictor of poor outcomes following CABG. However, following multiple regression analyses, inflammatory indices were not found to be an independent risk factor for poor postoperative outcomes. These findings contrast those of Dey et al.’s analysis in which they retrospectively evaluated poor outcomes following elective off-pump surgery. They concluded that only SII was an independent risk factor [5]. In the same study, the authors reported that the appropriate cut-off value for SII was 878,057×103/mm3; the cut-off value for SII in our study was 603.08×103/mm3. We believe that this variation in cut-off values is due to the varied definition of unfavorable postoperative outcomes in both studies. For example, Dey et al. defined “poor outcome” as more than one of the following: significant poor cardiac or cardiovascular event, duration of MV >24 hours, new-onset renal failure, sepsis, and death [5]. In our study, ST-segment elevation myocardial ischemia, IABP requirement, and cerebrovascular accident were added.

Higher SII values usually indicate strong inflammation and inadequate inflammatory response. Laboratory results from patients with preoperative high SII values typically reveal neutrophilia, lymphopenia, and thrombocytosis. Myocardial dysfunction and delay in postoperative recovery may be observed due to hyperactivation of the inflammatory response [11]. In addition, it has been reported that increased inflammation and oxidative stress are associated with unfavorable outcomes in cardiovascular disease [13]. Furthermore, activation of platelets, which are involved in hemostasis and the synthesis of adhesion molecules, causes complications in the postoperative period, such as stroke and myocardial infarction [8,13]. Therefore, our findings suggest that high SII and dNLR values indicating increased hyperinflammatory response may lead to poor postoperative clinical outcomes in patients undergoing CABG. However, the mechanism of the relationship between high preoperative inflammatory indices and poor clinical outcomes remains unclear in the literature; therefore, more research is needed to elucidate the underlying mechanism.

Other important inflammatory indices (CRP/Alb, CRP/L, and PLR) analyzed in our study were not statistically significant in univariate analysis; a significant difference was detected only in MPR value. In their study on the impact of inflammatory indices in predicting saphenous vein graft insufficiency following CABG, Oksuz et al. [14] found that high CRP level was a predictive factor. In addition, CRP and PLR levels were found to be predictive of 30-day major postoperative complications following isolated tricuspid valve surgery [11]. Aksoy et al. [15] reported that a high CRP/Alb ratio is a reliable marker for predicting the development of AF following CABG. In these studies, elevation of the acute phase reactant CRP during a hyperinflammatory response was associated with poor prognosis in cardiac surgery patients. Interestingly, MPR, but not MPV, differed significantly between the groups in our study. It has been reported in recent years that MPV, which plays an important role in the pathogenesis of atherosclerosis, is a prognostic factor associated with mortality in heart disease [16]. A recent study reported that high MPR values are predictors of poor outcomes after CABG [17]. In our study, high MPR values were found to be associated with unfavorable outcomes following univariate analysis; however, following multivariate analysis, after controlling for other variables, MPR was not found to be an independent predictor. Based on these findings, using inflammatory indices to predict unfavorable postoperative outcomes following CABG procedures and modifying options may help to mitigate unwanted postoperative outcomes. Nevertheless, our findings need to be interpreted with caution, since poor outcomes including mortality in cardiac surgery, particularly in CABG patients, may vary based on many perioperative factors such as İCU duration of stay, atrial fibrillation, neurological impairment, acute renal injury, and dialysis [18-20]. Previous studies identified many risk factors that might be associated with increased mortality including the use of İABP, increased cardiopulmonary bypass and aortic cross-clamp time, elevated preoperative creatinine levels exceeding 0.4 mg/dl, and patients over the age of 65 [20]. In addition, Rubino et al. [21] and Cakalagaoglu et al. [22] reported that technical failures during surgery and early graft occlusion, respectively, might contribute to early mortality after isolated CABG in low-risk patients. Furthermore, inadequate myocardial protection, particularly in patients with severe diffuse coronary artery disease, could lead to unfavorable outcomes after cardiac surgery [21]. Consistent with these studies, patients with poor outcomes in our cohort were frail, experienced prolonged aortic cross-clamp and cardiopulmonary bypass times, had an increased incidence of pulmonary complications, required the use of IABP more frequently, and had an extended length of stay in the ICU, rendering them more susceptible to complications and early mortality.

Strength and limitations

One of the major limitations of the study is being a retrospective observational study which may introduce inherent biases and limitations regarding data collection. Although we adjusted for various factors known to influence 30-day mortality and major postoperative complication rates after isolated CABG, the retrospective nature of this study may have masked potentially unknown confounding factors, thus our results should be interpreted with caution. In addition, only short-term outcomes were assessed due to the short follow-up period of 30 days after surgery. Furthermore, it is a single-center study with a relatively small sample size, which might limit the diversity of patients and the generalizability of the findings to other healthcare settings or populations. However, we performed a sample size calculation for statistical power analysis, which provided sufficient statistical power. Moreover, we included both on-pump and off-pump cases in this study, which may introduce a potentially confounding factor regarding the systemic inflammatory response induced by cardiopulmonary bypass during on-pump procedures. Nevertheless, the logistic regression analysis revealed no association between the type of surgery and poor outcomes. The most important strength of our study is that we included a comprehensive assessment of multiple inflammatory indices and biomarkers, which allowed for a more thorough understanding of the relationship between inflammation and postoperative outcomes after CABG procedures.

## Conclusions

Our study suggests that inflammatory indices, including SII, NLR, and dNLR, along with WBC and neutrophil counts, have the potential to serve as accessible markers for predicting poor outcomes following CABG procedures. These parameters may provide valuable insights into the potential impact of inflammatory indices on postoperative prognosis. However, given the limitations of our study, further research with a larger patient population and long-term outcomes after surgery with a comprehensive evaluation of all possible factors influencing unfavorable outcomes is required to validate and refine the use of these markers in clinical practice.
